# Naturally Derived Terpenoids Targeting the 3D^pol^ of Foot-and-Mouth Disease Virus: An Integrated In Silico and In Vitro Investigation

**DOI:** 10.3390/v16071128

**Published:** 2024-07-14

**Authors:** Natjira Mana, Sirin Theerawatanasirikul, Ploypailin Semkum, Porntippa Lekcharoensuk

**Affiliations:** 1 Department of Microbiology and Immunology, Faculty of Veterinary Medicine, Kasetsart University, Bangkok 10900, Thailand; natjira.ma@ku.th (N.M.); fvetpls@ku.ac.th (P.S.); 2 Department of Anatomy, Faculty of Veterinary Medicine, Kasetsart University, Bangkok 10900, Thailand

**Keywords:** foot-and-mouth disease virus (FMDV), terpenoids, FMDV 3D^pol^, antiviral activity

## Abstract

Foot-and-mouth disease virus (FMDV) belongs to the *Picornaviridae* family and is an important pathogen affecting cloven-hoof livestock. However, neither effective vaccines covering all serotypes nor specific antivirals against FMDV infections are currently available. In this study, we employed virtual screening to screen for secondary metabolite terpenoids targeting the RNA-dependent RNA polymerase (RdRp), or 3D^pol^, of FMDV. Subsequently, we identified the potential antiviral activity of the 32 top-ranked terpenoids, revealing that continentalic acid, dehydroabietic acid (abietic diterpenoids), brusatol, bruceine D, and bruceine E (tetracyclic triterpenoids) significantly reduced cytopathic effects and viral infection in the terpenoid-treated, FMDV-infected BHK-21 cells in a dose-dependent manner, with nanomolar to low micromolar levels. The FMDV minigenome assay demonstrated that brusatol and bruceine D, in particular, effectively blocked FMDV 3D^pol^ activity, exhibiting IC50 values in the range of 0.37–0.39 µM and surpassing the efficacy of the antiviral drug control, ribavirin. Continentalic acid and bruceine E exhibited moderate inhibition of FMDV 3D^pol^. The predicted protein–ligand interaction confirmed that these potential terpenoids interacted with the main catalytic and bystander residues of FMDV 3D^pol^. Additionally, brusatol and bruceine D exhibited additive effects when combined with ribavirin. In conclusion, terpenoids from natural resources show promise for the development of anti-FMD agents.

## 1. Introduction

Foot-and-mouth disease virus (FMDV) is one of the most significant infectious agents, causing considerable implications for the livestock industry, particularly cloven hoof animals [1]. This virus comprises a positive-sense, single-stranded RNA genome and belongs to the *Picornaviridae* family. FMDV is categorized into the following seven serotypes: A, O, C, Asia-1, SAT-1, SAT-2, and SAT-3, each with various subtypes [2]. Controlling FMDV presents significant challenging due to its serotype-specific nature, requiring the development and administration of serotype-tailored vaccines. However, the current vaccines lack cross-protection against different serotypes [3].

The existing FMDV vaccines are generally serotype-specific, posing a significant challenge in regions where multiple serotypes are prevalent. Consequently, a supportive antiviral strategy to offer broader protection against FMDV and prevent widespread outbreaks is necessary. The FMDV 3D^pol^, or RNA-dependent RNA polymerase (RdRp), is a crucial target, owing to its role in catalyzing genomic RNA replication and gene transcription. RNA synthesis is initiated by the viral VPg protein primer [4]. Inhibition or interference with this enzyme may result in impaired or stopped viral RNA replication, potentially reducing the numbers of the virus progeny. Examples of such inhibitors include remdesivir, which was utilized in emergency cases of COVID-19 [5], and sofosbuvir, which has been proven to be clinically effective in treating the hepatitis C virus [6]. Therefore, targeting the 3D^pol^ or RdRp of FMDV presents an intriguing avenue for developing antiviral drugs to control this disease.

Terpenoids, constituting the largest group of natural products, are natural chemical compounds with diverse structures. As they are mainly derived from 2-C-methyl-D-erythriol 4-phosphate (MEP) or mevalonic acid (MVA [7,8]), terpenoids consist of an isoprene unit (C5) and are categorized based on the number of carbon units in their main structures—hemiterpenoids (C5), monoterpenoids (C10), sesquiterpenoids (C15), diterpenoids (C20), sesterterpenoids (C25), triterpenoids (C30), tetraterpenoids or carotenoids (C40), and polyterpenes (C > 40). Terpenoids are mostly present in the form of volatile oils within higher medicinal plants and are mainly found in the following medicinal plant groups: *Compositae*, *Ranunculaceae*, *Araliaceae*, *Oleaceae*, *Magnoliaceae*, *Lauraceae*, *Aristolochiaceae*, *Rutaceae*, etc. [9]. These naturally derived compounds exhibit various biological and pharmacological properties, including documented antiviral activity [7,9].

Our research aims to specifically target the FMDV 3D^pol^, exploring the potential of terpenoids as inhibitors. This investigation employed virtual screening of secondary metabolite terpenoids from available databases to identify compounds possessing the potential to target the active site of FMDV 3D^pol^. Subsequently, we evaluated the cytotoxicity and antiviral activity of these terpenoids using cell-based assays. Additionally, the minigenome assay was utilized to identify terpenoids with anti-FMDV 3D^pol^ potential, which are capable of inhibiting FMDV replication.

## 2. Materials and Methods

### 2.1. Virtual Screening

#### 2.1.1. Preparation of FMDV 3D^pol^ and Terpenoids

The FMDV 3D^pol^ protein was modeled as a macromolecule, following a previously established protocol [10]. To achieve the homology model construction, the PDB structure (code ID: 1wne.pdb) served as the template, and the 3D^pol^ sequence of FMDV serotype A A/TAI/NP05/2017 (NP05) [11] was utilized to create the 3D protein structure. This procedure was carried out using the SWISS-MODEL server (https://swissmodel.expasy.org/, accessed on 1 January 2022).

For the virtual screening, terpenoid structures were obtained from PubChem (https://pubchem.ncbi.nlm.nih.gov/; accessed on 1 December 2022) and the Plant Secondary Compounds (PSC) databases [12]. A total of 738 ligand structures were prepared and organized into mini libraries before rendering the virtual screening process. The ligands were evaluated based on various physicochemical properties, including cLogP, hydrogen bond acceptors (H-Acceptors), hydrogen bond donors (H-Donors), total surface area, and drug-likeness, according to Lipinski’s Rule of 5 (Ro5) [13]. This assessment was performed using Data Warrior Version 5.5.0 (www.openmolecules.org).

#### 2.1.2. Molecular Docking and Protein–Ligand Interaction

The predicted binding interactions between the macromolecule and ligands were performed using AutoDock Vina within PyRx software version 0.9.8 [14,15]. In this molecular docking procedure, the grid center was set at coordinates 15:26:15 (x:y:z), with a corresponding grid box size of 30 Å × 30 Å × 35 Å. This configuration effectively encompassed all active sites of the polymerase pocket of FMDV 3D^pol^ [16] (Figure S1), which includes critical amino acids, namely, Asp240, Asp245, Asp338, and Asp339, and associate residues.

Default docking parameters were employed, with an exhaustiveness setting of eight and the number of modes set to nine. The resulting complexes generated by the molecular docking process were ranked based on their binding affinities. To further investigate the protein–ligand interactions, the top-ranked complexes were analyzed using BIOVIA Discovery Studio Visualizer 2021. In subsequent sections, the selected ligands were evaluated for their cytotoxicity and antiviral activity.

### 2.2. Cells, Virus, and Compounds

#### 2.2.1. Cells and Viruses

In this study, baby hamster kidney cells (BHK-21, ATCC^®^, Manassas, VA, USA) were employed. The cells were maintained in minimum essential medium (MEM, Gibco™ Thermo Fisher Scientific Inc., Waltham, MA, USA) supplemented with 10% fetal bovine serum (FBS, Gibco, Thermo Fisher Scientific Inc., Waltham, MA, USA), 2 mM L-glutamine (Invitrogen™, Carlsbad, CA, USA) and 1% antibiotic-antimycotic (Invitrogen™, Carlsbad, CA, USA).

The FMDV serotype A [A/TAI/NP05/2017 (NP05)] was titrated following the previous study [17] and had a viral titer of 1 × 10^7.5^ TCID50/mL, as determined using the Reed–Muench method [18]. The virus was stored in aliquots at −80 °C until it was ready for use.

#### 2.2.2. Terpenoid Compounds

Terpenoid compounds from ChemFaces (Wuhan, China) were selected using virtual screening based on RdRp of FMDV. The focused compounds were dissolved in dimethyl sulfoxide (DMSO, Sigma-Aldrich, St. Louis, MO, USA) to achieve a final stock concentration of 10 mM. The dissolved compounds were stored at −20 °C in preparation for cell-based assays.

### 2.3. Cytotoxicity Assay

To assess the impact of the compounds on cell viability, we conducted a cytotoxicity assay. BHK-21 cells were seeded at a concentration of 2.0 × 10^5^ cells/mL in a 96-well plate and incubated overnight at 37 °C with 5% CO_2_. On the following day, the compounds underwent a two-fold serial dilution in MEM to produce the final concentrations of 100, 50, 25, 12.5, 6.25, 3.12, 1.56, and 0.78 µM before adding to the culture media. As controls, media with and without 0.01% dimethyl sulfoxide (DMSO) were included.

The terpenoid-treated cells were incubated at 37 °C for 24 h. Subsequently, we evaluated cell viability using the Cell Counting Kit (CCK-8, TargetMol, Boston, MA, USA). Ten microliters of CCK-8 solution were added to each well and further incubated at 37 °C for 2 h. The optical density (OD) of the cell supernatants was measured at the wavelength 450 nm using a microplate reader (Synergy H1 Hybrid Multi-Mode Reader, BioTek^®^, Winooski, VT, USA) to quantify the formazan product (WST-8) generated by viable cells. We used the obtained OD values to determine the concentration at which cytotoxicity reached 50% (CC50) employing GraphPad Prism, version 10.1.1.

### 2.4. Antiviral Activity

To determine antiviral activity, BHK-21 cells were seeded at a concentration of 2.0 × 10^5^ cells/mL in a 96-well plate, following the overnight incubation process mentioned earlier. Subsequently, the cells were inoculated with FMDV at a concentration of 10 TCID50/mL and incubated at 37 °C for 2 h to facilitate virus absorption. Subsequently, the excess viruses were removed, and each well was rinsed twice with Rinse saline solution. Terpenoid compounds were subjected to serial ten-fold dilutions in MEM with 2% FBS, resulting in the final concentrations of 100, 50, 25, 10, 1, 0.5, and 0.1 µM, covering a range of 50% or 10% cytotoxicity concentrations (CC50 or CC10) before adding each dilution into duplicate wells. The terpenoid-treated, FMDV-infected BHK-21 cells were then incubated at 37 °C for 24 h. Ribavirin was used as a positive drug control, and 0.01% DMSO served as the negative control.

### 2.5. Cytopathic Effect (CPE) Inhibition Assay

Cytopathic effects (CPE) in FMDV-infected BHK-21 cells were evaluated by observing the rounding-up of infected cells and cell lysis characteristics, typically occurring within 25–26 h following FMDV infection. This experiment was aimed to determine whether terpenoid-treated, FMDV-infected BHK-21 cells exhibited reduced CPE in a dose-dependent manner. Following the incubation, the attached cells were fixed with cold absolute methanol at room temperature for 15 min, and the plates were allowed to dry. Subsequently, the cells were stained with a 0.5% crystal violet solution, and the stained cells were observed using a phase-contrast inverted microscope (Olympus IX73, Tokyo, Japan). Images of cell viability areas were recorded by photography, which were further analyzed using CellProfiler Version 4.2.5. For each tested compound, the 50% effective concentration (EC50) was calculated using GraphPad Prism, version 10.1.1.

### 2.6. Immunoperoxidase Monolayer Assay (IPMA)

IPMA was carried out according to the protocol described previously [19] to confirm antiviral activity in addition to the CPE inhibition assay. Duplicate plates were performed independently to detect FMDV antigens in the terpenoid-treated, FMDV-infected BHK-21 cells at 18 h post-inoculation (hpi). After cell fixing with absolute methanol, the cells were washed with PBS containing 0.05% Tween (PBST) and then treated with 50 µL/well of a blocking buffer (BlockPRO™ 1 Min Protein-Free Blocking Buffer, Taipei, Taiwan) for 1 min. Subsequently, the cells were washed with PBST and incubated with a single-chain variable fragment with Fc fusion antibody (scFv-Fc) specific to 3ABC of FMDV (1:200) [20] at 37 °C for 1 h.

Following the primary antibody incubation, the cells were washed with PBST and then incubated with HRP-conjugated protein G (EMD Millipore Corporation, Temecula, CA, USA) at 37 °C for another hour. The presence of the FMDV antigen in the infected cells was visualized using DAB substrate (DAKO, Santa Clara, CA, USA), resulting in a dark-brown cytoplasmic staining. The staining for each compound was recorded and the positive-infected cells were analyzed using CellProfiler Version 4.2.5. The 50% effective concentration (EC50) was calculated using GraphPad Prism, version 10.1.1.

### 2.7. Reverse Transcription-Quantitative Polymerase Chain Reaction (RT-qPCR)

In our investigation, we performed RT-qPCR to evaluate viral RNA levels in terpenoid-treated, FMDV-infected BHK-21 cells and compared them with viral-infected and normal cell controls. The experimental workflow included the key steps described below.

BHK-21 cells were initially seeded at a density of 2.0 × 10^5^ cells/mL in a 24-well plate and incubated overnight. Subsequently, these cells were inoculated with FMDV at a concentration of 10 TCID50/well and incubated at 37 °C for 2 h. Following viral absorption, the cells were washed with Rinse saline solution. The cells were then treated with the test compounds at serial concentrations and incubated at 37 °C for 24 h, as previously described [16,21]. After treatment, the cells and supernatants were harvested, and total RNA was isolated using Trizol^TM^ reagent (Thermo Fisher Scientific Inc., Waltham, MA, USA), followed by the Direct-zol^TM^ RNA MiniPrep kit (Zymo Research Corporation, Tustin, CA, USA), according to the manufacturer’s guidelines.

cDNA was synthesized from 1 µg of the total RNA using RevertAid reverse transcriptase (Thermo Fisher Scientific Inc., Waltham, MA, USA). For viral load quantification, random hexamers (Invitrogen™, Carlsbad, CA, USA) were employed as primers to generate the first stand cDNA, while a specific primer targeting the negative-stranded RNA within the 3D^pol^ coding sequence (Table 1) was used for cDNA synthesis to quantify negative-stranded RNA levels, as described in our prior study [16,21]. The primers used to amplify 5′UTR of FMDV cDNAs for viral load quantification were FMDV-5′UTR_F and FMDV-5′UTR_R and the 3D^pol^ region for negative strand quantification were FMDV-3DF and FMDV-3DR, respectively (Table 1).

The qPCR was performed using iTaq Universal SYBR Green Supermix (Bio-Rad Laboratories, Hercules, CA, USA) and a C1000 Touch thermal cycler (Bio-Rad Laboratories), with the PCR conditions in accordance with the protocols established in our previous study [16,21]. For viral load quantification, Ct values were obtained through PCR amplification, and sample viral copy numbers were determined using a standard curve generated from a plasmid carrying FMDV 5′UTR, with ten-fold serial dilutions ranging from 10^−2^ to 10^−7^ plasmid molecules/µL. The viral loads in the terpenoid-treated, FMDV-infected cells were compared to those in virus infection and cell controls. In cases of negative-stranded RNA quantification, data were normalized based on delta Ct values [16,21].

### 2.8. Determination of Anti-FMDV 3D^pol^ Activity Using an FMDV Minigenome Assay

In our investigation, we aimed to determine the potential inhibitory effects of terpenoids on FMDV 3D^pol^, a pivotal viral protein crucial for viral replication. This inquiry was carried out through a cell-based FMDV minigenome assay, following the method described by Semkum et al. in 2021 [10]. To briefly outline the assay, tri-transfection was performed, involving plasmid pKLS3_GFP, designed for GFP expression and containing the FMDV O189 5′ and 3′ UTRs, along with the following two indispensable helper plasmids: pCAGGS_T7, which contained T7 RNA polymerase, and pCAGGS_P3, encompassing the FMDV P3 region. BHK-21 cells were seeded at a density of 2.0 × 10^5^ cells/mL in a 96-well plate. Subsequently, a final volume of 50 µL per well of plasmids mixed with Fugene^®^ HD and Opti-MEM™ I Reduced-Serum Medium was incubated for 15 min at room temperature. The transfection mixtures were then replaced with serially diluted terpenoid compounds in Opti-MEM™ I Reduced-Serum Medium. The plate containing the transfected cells was incubated at 37 °C for 24 h. For negative controls, 0.01% DMSO and empty plasmids were used. After transfection, the GFP expression of the transfected cells was documented, and the fluorescent signals were quantified in comparison to the control samples. The inhibitory concentration (IC50) is the concentration of a terpenoid at which the activity of 3D^pol^ is reduced by 50%, compared to that of the DMSO controls.

### 2.9. Antiviral Combination Assay of Potential Terpenoids

BHK-21 cells were seeded in 96-well plates at a density of 2.0 × 10^5^ cells/mL. Following two hours of FMDV infection, the media were removed and replaced with MEM supplemented with 2% FBS containing various combinations of the potential terpenoids and the control drug ribavirin. These combinations were prepared in a 6 by 6 matrix format, with serial dilutions of each compound along the axes. DMSO was used as a vehicle control. All combinations were tested in duplicate. After treatment, cells were incubated at 37 °C for 24 h. Cell viability was then assessed using the Cell Counting Kit-8 (CCK-8, TargetMol, USA), as mentioned earlier. The synergistic effects of the drug combinations were analyzed using SynergyFinder version 3.0, employing the ZIP and HSA methods, as described by Ianevski et al. in 2022 [22]. This methodology allows for the evaluation of potential synergistic, additive, or antagonistic effects between the selected terpenoids and ribavirin in inhibiting FMDV replication.

## 3. Results

### 3.1. Virtual Screening of Terpenoid Compounds

In our investigation, we identified terpenoids with the potential to inhibit FMDV 3D^pol^. We utilized virtual screening to model the molecular interactions between viral proteins and these compounds. The predicted FMDV 3D^pol^ in this study was based on the sequence of FMDV serotype A, A/TAI/NP05/2017 (NP05) [11]. We positioned the active site residues of FMDV 3D^pol^, namely Asp240, Asp245, Asp338, and Asp339, within the polymerase pocket of 3D^pol^. Additionally, we conducted virtual screening in the area covering residues Pro44, Pro169, and Met296, which are crucial residues involved in the 3D^pol^ functionality [23].

During the virtual screening of 738 terpenoid ligands, we determined the best-ranked ligands, based on binding affinity, which exhibited values ranging from −10.5 to −4.6 kcal/mol. Subsequently, we set a cutoff at −6.0 kcal/mol for further analysis of protein–ligand interactions. We subjected them to evaluate their physicochemical analysis based on Lipinski’s Rule of 5 criteria [13], which includes a molecular weight (MW) of <500 Da, hydrogen bond donors (H-donors) ≤ 5, hydrogen bond acceptors (H-acceptors) ≤ 10, a calculated logarithm of the octanol/water partition coefficient (cLogP) < 5, and a molar refractivity within the range of 40 to 130. We found that some of the terpenoids violated the Ro5. The 32 top-ranked ligands were selected as focused compounds for further evaluation in cell-based assays, and the compounds are listed in Table S1.

### 3.2. Cytotoxicity Assay to BHK-21 Cells

Following the initial screening of the 32 terpenoid compounds, which highlighted the top-performing candidates from our virtual screening, we proceeded to assess their cytotoxicity on BHK-21 cells. The results revealed that the majority of terpenoids exhibited minimal toxicity to non-toxicity toward BHK-21 cells, with cell viability ranging from approximately 50% to 100%. The CC50 values of the low to non-toxic terpenoids spanned from 46.51 ± 1.67 µM to over 100 µM for these compounds (Table S2). Additionally, three triterpenoids displayed a moderate level of cytotoxicity. Specifically, brusatol exhibited a CC50 value of 18.54 ± 1.27 µM, bruceine D showed a CC50 of 4.51 ± 0.68 µM, and bruceine E had a CC50 of 49.65 ± 1.70 µM (Table 2). Most terpenoids were less toxic, possessing very high CC50 values. Detailed information regarding cell morphology and the corresponding cytotoxicity values is present in Table S2.

### 3.3. Antiviral Activity of Terpenoids

Subsequently, we investigated the antiviral efficacy of terpenoids against FMDV infection, considering their impact on BHK-21 cell viability. In the initial cell-based screening, all 32 terpenoids were evaluated, and it was observed that 5 of them exhibited antiviral activities against FMDV infection. These active terpenoids were identified as brusatol, bruceine D, bruceine E, continentalic acid, and dehydroabietic acid. In contrast, the remaining 27 terpenoids did not demonstrate any antiviral effects.

**Figure 1:**
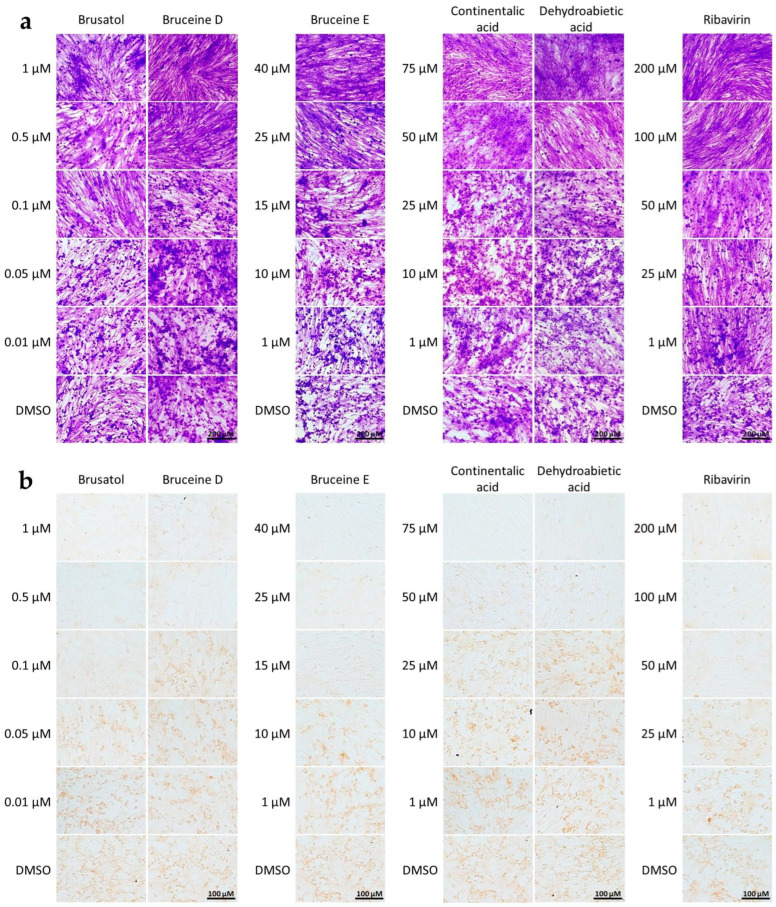
Cell-based antiviral activity of terpenoids against FMDV infection in BHK-21 cells. (**a**) Cytopathic effect (CPE) inhibition assay: The cells were stained with 0.5% crystal violet solution, showing rounding-up and clustering characteristics of infected cells, while non-infected cells exhibit elongated, spindle-shaped morphology similar to those in the control. Scale bars represent 200 µm. (**b**) Detection of FMDV-infected cells determined by IPMA, revealing brown staining of FMDV antigens in the cytoplasm, whereas non-infected cells do not show positive signals. DMSO and ribavirin serve as vehicle (no drug, virus-infected cell) control and drug control, respectively. Scale bars represent 100 µm.

The cytopathic effect (CPE) of FMDV infection was characterized by the rounding-up of infected cells and the diffuse cell clusters and aggregation, as observed in crystal violet staining. When these cells were treated with the aforementioned five terpenoids, a dose-dependent reduction in CPE was observed (Figure 1a). Furthermore, we confirmed their antiviral potency using IPMA with antibodies specific to FMDV 3ABC antigens in the terpenoid-treated, FMDV-infected cells. The FMDV-infected cells treated with the five terpenoids exhibited reduced brown staining compared to the positive staining seen in non-treated, FMDV-infected cells (DMSO) (Figure 1b).

**Figure 2:**
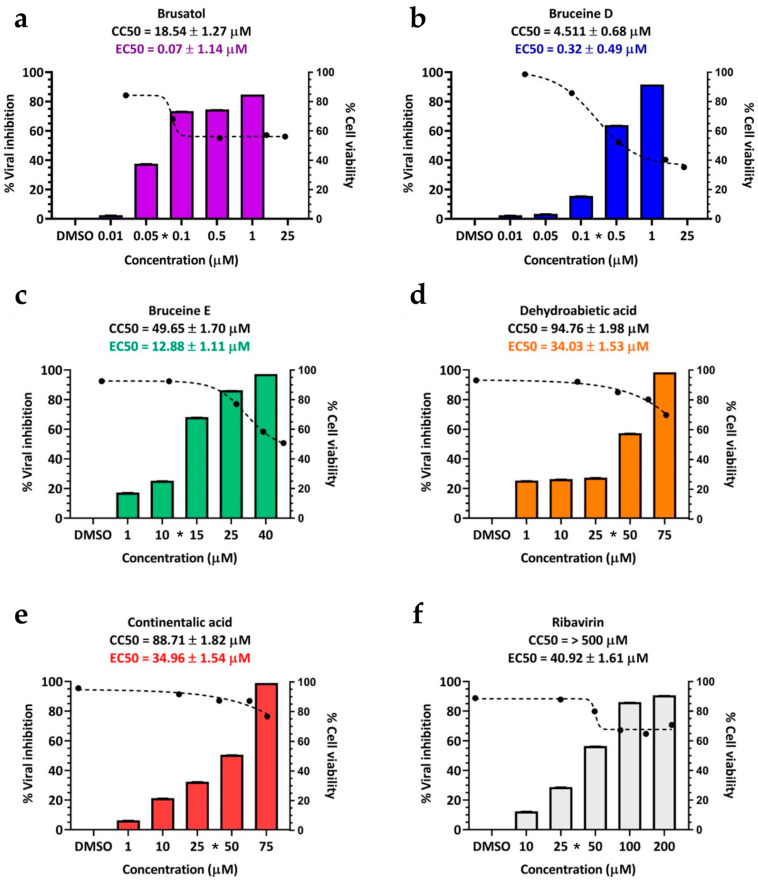
Cell cytotoxicity and antiviral activity of selected terpenoids. The cytotoxicity and antiviral activity were assessed using CCK-8 and IPMA, respectively. The black dashed lines represent dose-dependent cytotoxicity concentrations, while the bar charts depict the inhibitory effect on FMDV infections. The selected terpenoids include (**a**) brusatol, (**b**) bruceine D, (**c**) bruceine E, (**d**) continentalic acid, and (**e**) dehydroabietic acid. (**f**) Ribavirin serves as an antiviral drug control. Asterisks (*) on the *x*-axis of each terpenoid graph indicate the effective concentrations.

The 50% effective concentration (EC50) values for these terpenoids as determined by IPMA were as follows: 0.07 ± 1.14 µM for brusatol, 0.32 ± 0.49 µM for bruceine D, 12.88 ± 1.11 µM for bruceine E, 34.96 ± 1.54 µM for continentalic acid, and 34.03 ± 1.53 µM for dehydroabietic acid. In this study, ribavirin served as the drug control, with an EC50 value of 40.92 ± 1.61 µM (Table 2 and Figure 2). Interestingly, two structurally related terpenoids, abietic acid, and kaurenoic acid, did not exhibit antiviral activity (Table 2).

### 3.4. Viral Load Quantification Using RT-qPCR

To evaluate viral replication within the terpenoid-treated, FMDV-infected cells, as described in the aforementioned antiviral activity assay, we conducted a two-dimensional assessment. This assessment encompassed the quantification of viral copy numbers utilizing FMDV 5′UTR sequence detection, as well as the production of negative-stranded RNA attributed directly to FMDV 3D^pol^ function.

**Figure 3:**
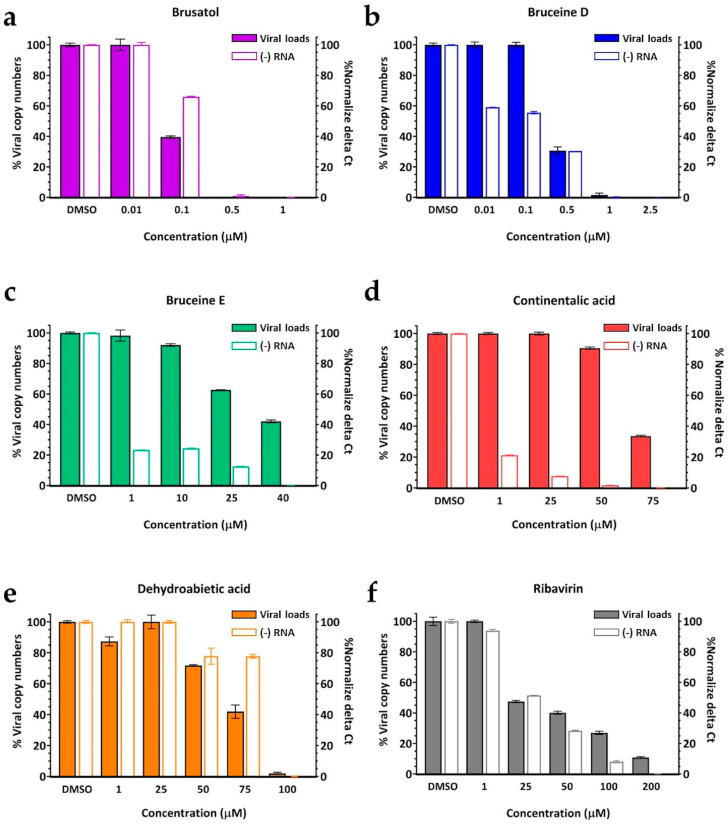
Viral load detection in terpenoid-treated, FMDV-infected cells using RT-qPCR. Viral loads were reported as % viral copy numbers (colored bars, right *y*-axes), and negative-stranded RNA production was quantitated as % normalized Ct (clear bars, left *y*-axes). The selected terpenoids include (**a**) brusatol, (**b**) bruceine D, (**c**) bruceine E, (**d**) continentalic acid, and (**e**) dehydroabietic acid. (**f**) Ribavirin is the antiviral drug control.

The results demonstrated that all five terpenoids exhibited the ability to decrease viral loads and replications in a dose-dependent manner (Figure 3). Brusatol and bruceine D completely inhibited FMDV within the concentration ranges of 0.5 to 1 µM and 1 to 2.5 µM, respectively. In contrast, bruceine E displayed moderate potency against FMDV infection. Continentalic acid demonstrated moderate inhibition in viral load, with a noticeable reduction in the negative-stranded RNA production at a concentration of 25 µM. The RT-qPCR results align with the findings through the assessments of CPE and IPMA, in which four terpenoids exhibited superior viral inhibition compared to ribavirin, with the exception of dehydroabietic acid (Figure 3). Dehydroabietic acid exhibited low to moderate inhibition of both viral load and negative-stranded RNA production. In fact, dehydroabietic acid demonstrated effective inhibition of viral load at a high dose but did not show a comparable impact on the negative-stranded RNA at 75 µM.

### 3.5. Inhibition of FMDV 3D^pol^ Using an FMDV Minigenome Assay

**Figure 4:**
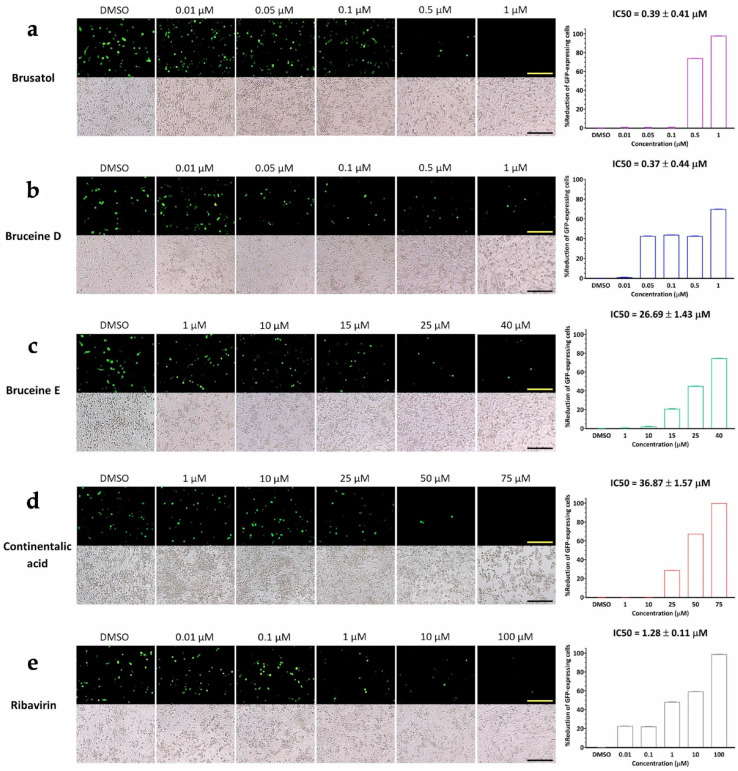
Inhibition of FMDV 3D^pol^ by the selected terpenoids evaluated using the FMDV minigenome assay. The selected terpenoids include (**a**) brusatol, (**b**) bruceine D, (**c**) bruceine E, and (**d**) continentalic acid. (**e**) Ribavirin is the antiviral drug control. The upper panels show the GFP expression driven by FMDV 3D^pol^ in BHK21 cells. Terpenoids could inhibit FMDV 3D^pol^ activity in a dose-dependent manner, as shown by the reduction of GFP-expressing cells. The lower panel shows bright-field images of the terpenoid-treated, plasmids-transfected cells in each well corresponding to the upper dark-field images. Scale bars represent 200 µm. The % reduction of GFP-expressing cells is depicted in the bar chart.

Our investigation was centered on assessing the potential influence of candidate compounds on FMDV 3D^pol^ function by employing the FMDV minigenome system with the GFP expression to quantify the levels of FMDV 3D^pol^ activity. This was achieved through the transfection of three plasmids into BHK-21 cells. The results of the transfection revealed that brusatol and bruceine D exhibited the capability to inhibit 3D^pol^, resulting in diminished GFP expression within BHK-21 cells at the nano- to micromolar range (Figure 4). Their respective IC50 values were measured at 0.39 ± 0.41 µM and 0.37 ± 0.44 µM for brusatol and bruceine D, respectively. In contrast, bruceine E and continentalic acid required slightly higher concentrations to inhibit 3D^pol^, with IC50 values of 26.69 ± 1.43 µM and 36.87 ± 1.57 µM, respectively. However, dehydroabietic acid, which exhibited moderate viral inhibition, also required a high dose to disrupt the function of 3D^pol^ (IC50 > 50 µM in accordance with the findings of negative-stranded RNA production from the RT-qPCR analyses (Figure 3). The transfected cells expressing GFP treated with a serial concentration of terpenoids showed no sign of toxicity from either treatment or transfection, as presented in the lower panel of Figure 4.

### 3.6. Protein–Ligand Interaction

We conducted a detailed exploration of the molecular interactions between FMDV 3D^pol^ and terpenoids, focusing on the binding interactions at the lowest energy conformations for effective viral inhibition. The terpenoids, with their binding affinities, were as follows: brusatol (−8.2 kcal/mol), bruceine D (−8.1 kcal/mol), bruceine E (−7.9 kcal/mol), continentalic acid (−6.0 kcal/mol), and dehydroabietic acid (−6.8 kcal/mol). These ligands were selected based on their binding energies and efficacy in inhibiting viral activity.

**Figure 5:**
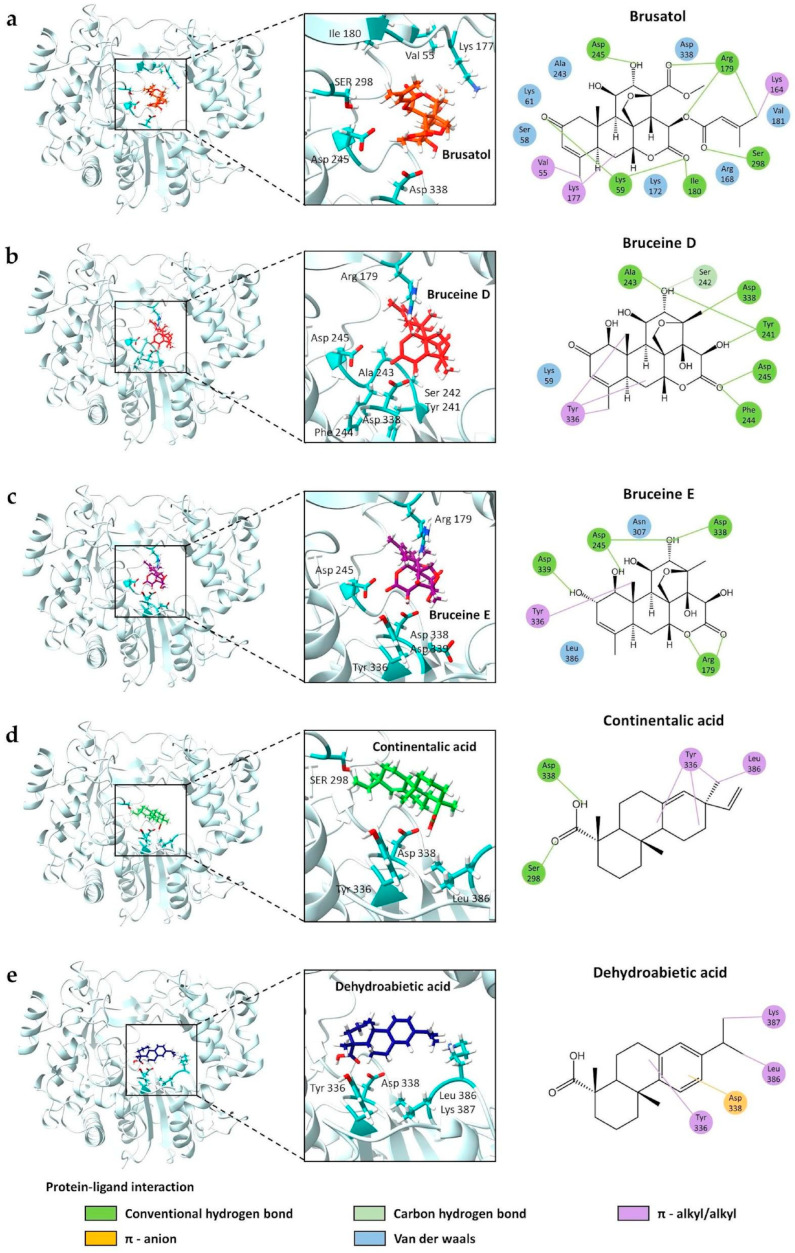
Interaction between FMDV 3D^pol^ protein and selected terpenoids using AutoDock Vina via PyRx software. (**a**) Brusatol, (**b**) bruceine D, (**c**) bruceine E, (**d**) continentalic acid, and (**e**) dehydroabietic acid. The 3D structures show terpenoids reaching the enzymatic pocket of FMDV 3D^pol^. The right panel demonstrates specific ligand–protein interactions in 2D structure.

Active site residues of FMDV 3D^pol^, particularly Pro44, Pro169, and Met296 in the finger and palm domains, play a crucial role in interactions between 3D^pol^ and RNA. However, none of the terpenoids interacts with them. At least two of the three catalytic residues, including Asp245, Asp338, and Asp339, in the enzymatic pocket [23], form hydrogen bonds with brusatol, bruceine D, and bruceine E (Figure 5a–c), consistent with the results of both cell-based and FMDV minigenome assays. Interactions of the terpenoids with the catalytic bystander residues may assist the binding stability of the compounds. In addition, bruceine E, despite exhibiting lower affinity than its derivatives, interacts with the three main catalytic residues (Figure 5c).

Continentalic acid, with a binding affinity of −6.0 kcal/mol, formed a hydrogen bond solely with the Asp338 residue in the catalytic site, while dehydroabietic acid engaged in a π-anion interaction (Figure 5d,e). Both continentalic acid and dehydroabietic acid exhibited low to moderate antiviral activities, which required higher doses than the three triterpenoids in the cell-based and FMDV minigenome assays, consistent with the findings from molecular docking.

### 3.7. Brusatol and Bruceine D Enhance the Antiviral Activity of Ribavirin

We further explored the combined effects of the potent terpenoids brusatol and bruceine D, which showed promising antiviral activity and inhibition of FMDV 3D^pol^. These compounds were evaluated alongside the positive control drug, ribavirin. The antiviral activity of various drug combinations was assessed using a CCK-8 assay. Our findings indicate that the combination of brusatol or bruceine D with ribavirin demonstrated enhanced efficacy against FMDV post-infection compared to single-drug treatments.

Using SynergyFinder, we calculated interaction scores using multiple models. In the mixture of brusatol and ribavirin, we observed ZIP (4.539) and HSA (9.570) scores, with the most effective combination area at brusatol 0.02–0.1µM and ribavirin 1–50 µM. These results suggest that brusatol could enhance the antiviral activity of ribavirin through an additive effect. For the bruceine D and ribavirin composition, we observed ZIP (−5.859) and HSA (−0.827) scores, with the most effective combination area at bruceine D 0.01–0.5 µM and ribavirin 10–100 µM. These results indicate positive interactions between each terpenoid and ribavirin (Figure S2). While not reaching the threshold for synergy, these additive effects suggest that combining these compounds with ribavirin may offer improved therapeutic strategies for combating FMDV infections.

## 4. Discussion

Our investigation of terpenoids as potential inhibitors of FMDV 3D^pol^ has yielded significant findings. Through virtual screening, we identified 32 terpenoids exhibiting promising binding affinities; however, some of them violated Lipinski’s Rule of 5 (Ro5) criteria. Many natural products (NPs) do not fall within the Ro5 parameters, referred to as the “parallel universe of Ro5 space” or “NP-Ro5 outliers” [24,25]. Most natural compounds violate at least two Ro5 criteria but can still elicit favorable biological properties and be considered “druggable” [25]. Notably, in the antiviral activity screening, five terpenoids—abietane diterpenoids, including dehydroabietic acid and continentalic acid, and triterpenoids, including brusatol, bruceine D, and bruceine E—demonstrated potent inhibition against FMDV. These terpenoids effectively reduced cytopathic effects and inhibited viral replication in infected cells, which was supported by RT-qPCR analysis, confirming their dose-dependent inhibition of viral replication.

The examination of the viral target protein using the FMDV minigenome assay revealed that brusatol and bruceine D exhibited the potent inhibition of FMDV 3D^pol^ activity at the nanomolar level, while the others showed effectiveness in the low micromolar range, except for dehydroabietic acid. This was substantiated by a noticeable decrease in GFP expression. Protein–ligand interaction analyses uncovered crucial binding affinities and interactions with the catalytic site residues of FMDV 3D^pol^.

In the terpenoid screening, we discovered that both diterpenoids and triterpenoids exhibited potent inhibition against FMDV. Diterpenoid structures consist of 20 carbon atoms (C20) or four isoprene units, whereas triterpenoids consist of 30 carbon atoms (C30) or six isoprene units. [7]. These compounds are present in various plant genera, including coffee and spices, pine trees (*Pinus densiflora*, *P. sylvestris*, *Abies grandis*), rosemary (*Rosmarinus officinalis*), oregano (*Origanum vulgare*), and *Andrographis paniculata*, or resin or bark extracts of conifers [8,9]. Tetracyclic triterpenoids, namely quassinoids, can be extracted from plants in the genus *Brucea* and the family *Simaroubaceae*, mainly found in Southeast Asia, including Thailand and northern Australia [26,27].

Brusatol has demonstrated antiviral efficacy by reducing HCV RNA levels post-infection and decreasing the expression of the Nrf2 protein, which plays a role in regulating the proliferation and metabolism of liver cancer cells [28]. Moreover, brusatol has been shown to reduce cell survival and promote cell death in hepatocellular carcinoma through autophagy via the PI3K/Akt/mTOR pathway [29]. Interestingly, it exhibits a synergistic effect with sorafenib, enhancing the inhibitory effects against HCV and demonstrating anti-liver cancer properties [28]. Sorafenib has recently been discovered to inhibit FMDV 3D^pol^ and the cellular c-RAF pathway [21]. Brusatol has been reported to have antiviral activity against tobacco mosaic virus (TMV) [30].

Bruceine D and E, tetracyclic triterpene quassinoids, are also found in the *Simaroubaceae* family including *Brucea javanica.* It has been reported that bruceine D exhibits antiviral activity against plant viruses, including TMV, potato virus Y (PVY), and cucumber mosaic virus (CMV) [31]. Recently, bruceine D has been shown to significantly inhibit ZIKV with an IC50 of 0.36 μM after infection using a stable ZIKV GFP-reporter virus system in Vero and BHK-DR cells [32]. Moreover, the mechanism of action of quassinoid extracts from *Eurycoma longifolia* has demonstrated that quassinoid 6α-HEL could reduce plaque formation of DENV-2 and bound to DENV-2 NS5-RdRp protein [33]. Herein, we explored the inhibitory effects of brusatol, and bruceine D, and E on FMDV 3D^pol^ (RdRp), which is suggested by our FMDV minigenome assay and protein–ligand interaction.

These tetracyclic triterpenoids (Figure S3), particularly brusatol, contain the main four rings, as follows: a cyclohexanone ring (A), two cyclohexane rings (B, C), a six-membered lactone ring (D), and a tetrahydrofuran ring (E) [26,34]. Bruceine D and E have substituents connected to the A-, C-, and D-rings, without the E-ring. There is a slight difference between bruceine D and E at the C2 position of the A-ring, with a carbonyl group for bruceine D and a hydroxyl group for bruceine E (Figure S3). In our predicted model, the C12 position of the C-rings of brusatol and bruceine E forms hydrogen bonds directly with Asp245 of FMDV RdRp catalytic triad, whereas bruceine D forms bonds between the carbonyl group at the C16 position of the D-ring, with FMDV RdRp Asp245 and the methyl group at the C13 position of the C-ring with FMDV RdRp Asp338 (Figure 5 and Figure S3). Therefore, the predicted molecular interactions confirmed that these quassinoids can bind to FMDV 3D^pol^ and reduce viral activity, providing valuable insights for the development of antiviral agents against foot-and-mouth disease. Nevertheless, functional analyses by mutations of the interactive residues in the RdRp catalytic pocket formed by the FMDV minigenome can reaffirm the important roles of these residues in binding with the terpenoid compounds and in RNA replication.

Another group of terpenoids, dehydroabietic acid and continentalic acid, are classified as tricyclic diterpenoids, aromatic abietane-type, similar to abietic acid, which exhibit a wide range of biological activities including antimicrobial, anti-tumor, or regenerative properties [35,36]. Among diterpene compounds, dehydroabietic acid has been evidenced for antiviral properties or antiviral action [7]. It demonstrated moderate activity against human herpes simplex virus-2 (HSV-2) but did not inhibit HSV-1 in in vitro assays on Vero cells, whereas abietic acid did not exhibit such activity [37], consistent with our findings on antiviral activity against FMDV infection. Additionally, there are reports indicating that synthetic derivatives at C18 and C19 positions of dehydroabietic acid could diminish titers of human cytomegalovirus (CMV), varicella-zoster virus (VZV) [38], HSV-1 [35], chikungunya virus (CHIKV), Zika virus (ZIKV), and dengue virus type 2 (DENV-2) [39]; however, specific mechanisms of abietane diterpenoids for the antiviral activity were not elucidated.

In the case of continentalic acid, previous studies have highlighted its anti-inflammatory and anti-arthritic activities, demonstrating a significant inhibition of the IL-1β-stimulated phosphorylation of p38, ERK1/2, and JNK protein kinases [40]. Regarding antimicrobial activity, continentalic acid and kaurenoic acid from *Aralia continentalis* have been shown to inhibit biofilm formation and exert bactericidal effects against *Streptococcus mutans* and methicillin-resistant *Staphylococcus aureus* [41,42]. However, antiviral activity has not been previously identified. In this study, we observed, for the first time, that continentalic acid exhibited inhibitory effects on the virus.

To elucidate the possible mechanisms of abietane diterpenoids, the structure–activity relationships (SARs) of these compounds are characterized by an ABC-ring system (Figure 5 and Figure S3), revealing functionalization not only on the A-ring carbons but also varying degrees of oxygenation on their B- and C-ring carbons, as well as carbon at positions 18–20, contributing to their diverse properties [43]. Notably, continentalic acid features an alkene group connected to the C-ring and a carboxylic group on the A-ring. However, to the best of our knowledge, this structural arrangement aids in occupying and interacting with Asp338, the key residue of the catalytic site of FMDV 3D^pol^. This structural feature positions continentalic acid as a more consistent inhibitor than dehydroabietic acid, as suggested by the results of molecular docking and FMDV 3D^pol^ inhibitory assay in our study.

## 5. Conclusions

In summary, our investigation on the antiviral activities of terpenoids involved virtual screening to narrow down secondary metabolites from plant sources, specifically targeting them to FMDV 3D^pol^. We first identified highly promising metabolites, including abietane diterpenoids and tetracyclic triterpenoids, demonstrating their potential to inhibit viral replication in a dose-dependent manner and reduce GFP expression driven by 3D^pol^ in the FMDV minigenome assay. Further research is needed to comprehensively understand their derivatives or related secondary metabolites and explore other synthetic modifications. This exploration is crucial for the potential use of these triterpenoids as lead compounds for non-nucleoside inhibitors (NNIs), thereby opening avenues for the development of antiviral strategies and therapeutic interventions against FMDV.

## Figures and Tables

**Table 1:** Details of primers used in this study.

Names	Procedure	Target	Objective	Sequences (5′ → 3′ Direction)
Random hexamers	cDNA synthesis	NA	Viral load quantification	NA ^1^
3D^pol^ gene specific primer		3D^pol^	Negative-stranded RNA quantification	AAGGGTTGATTGTTGACA
FMDV-5′UTRF	RT-qPCR	5′UTR	Viral load quantification	CTGTTGCTTCGTAGCGGAGC
FMDV-5′UTRR		5′UTR	Viral load quantification	TCGCGTGTTACCTCGGGGTACC
FMDV-3DF		3D^pol^	Negative-stranded RNA quantification	TAGAGCAGTAGATGTTG
FMDV-3DR		3D^pol^	Negative-stranded RNA quantification	ATGAACATCATGTTTGAGG

^1^ NA = Not applicable.

**Table 2:** Cytotoxicity and antiviral activity of terpenoids.

Terpenoids	Cytotoxicity (CC50 ^1^, µM)	Antiviral Activity (EC50 ^2^, µM)		
		CPE (Crystal Violet)	FMDV-Infected Cells (IPMA)	Viral Copy Number (RT-qPCR)
Triterpenoids				
Brusatol	18.54 ± 1.27	0.18 ± 0.27	0.07 ± 1.14	0.09 ± 1.04
Bruceine D	4.51 ± 0.68	0.25 ± 0.60	0.32 ± 0.49	0.42 ± 0.38
Bruceine E	49.65 ± 1.70	18.09 ± 1.26	12.88 ± 1.11	33.44 ± 1.52
Diterpenoids				
Abietic acid	99.25 ± 2.00	Not inhibit	ND	ND
Continentalic acid	88.71 ± 1.82	43.36 ± 1.64	34.96 ± 1.54	68.28 ± 1.83
Dehydroabietic acid	94.76 ± 1.98	41.73 ± 1.62	34.03 ± 1.53	64.55 ± 1.81
Kaurenoic acid	266.1 ± 2.42	Not inhibit	ND	ND
Drug control				
Ribavirin	>500	64.54 ± 1.81	40.92 ± 1.61	27.70 ± 1.44

ND = Not determined. ^1^ CC50: Half-maximal cytotoxic concentration, evaluated by CCK-8 assay. ^2^ EC50: Half-maximal effective concentration, determined using FMDV-infected BHK-21 cells. Values are expressed in micromolar (µM).

## Data Availability

No new data were created or analyzed in this study. Data are available upon request.

## Supplementary Materials

The following supporting information can be downloaded at https://www.mdpi.com/article/10.3390/v16071128/s1: Figure S1. Parameters of the grid box and grid center used for virtual screening in PyRx software. The grid center was set at coordinates 15:26:15 (x:y:z) with a grid box size of 30 Å × 30 Å × 35 Å, which effectively covered the active site pocket and associated residues of the FMDV 3D^pol^. Figure S2. Interaction effects of the combination of either brusatol or bruceine D and the drug control, ribavirin, were analyzed using SynergyFinder version 3.0. The interaction scores for each method are depicted for (a) brusatol with ribavirin and (b) bruceine D with ribavirin. Summary synergy scores are interpreted as follows: <−10 is antagonistic, −10 to 10 is additive, and >10 is synergistic. Figure S3: Chemical structures of (a) tetracyclic triterpenoids (brusatol, bruceine D, bruceine E) and (b) abietane diterpenoids (abietic acid, dehydroabietic acid, and continentalic acid); Table S1: Structural-based virtual screening and physicochemical properties of 32 top-ranked terpenoids. Table S2. Cytotoxicity level of terpenoids on BHK-21 cells using crystal violet staining.
