# Supplementary material for: Naturally Derived Terpenoids Targeting the 3Dpol of Foot-and-Mouth Disease Virus: An Integrated In Silico and In Vitro Investigation
Source: Viruses. 2024 Jul 14;16(7):1128. doi: 10.3390/v16071128 (PMC11281344; doi:10.3390/v16071128)
Supplement: Supplementary file 1 [file viruses-16-01128-s001.zip › viruses-3050528-supplementary.pdf]

Supplementary

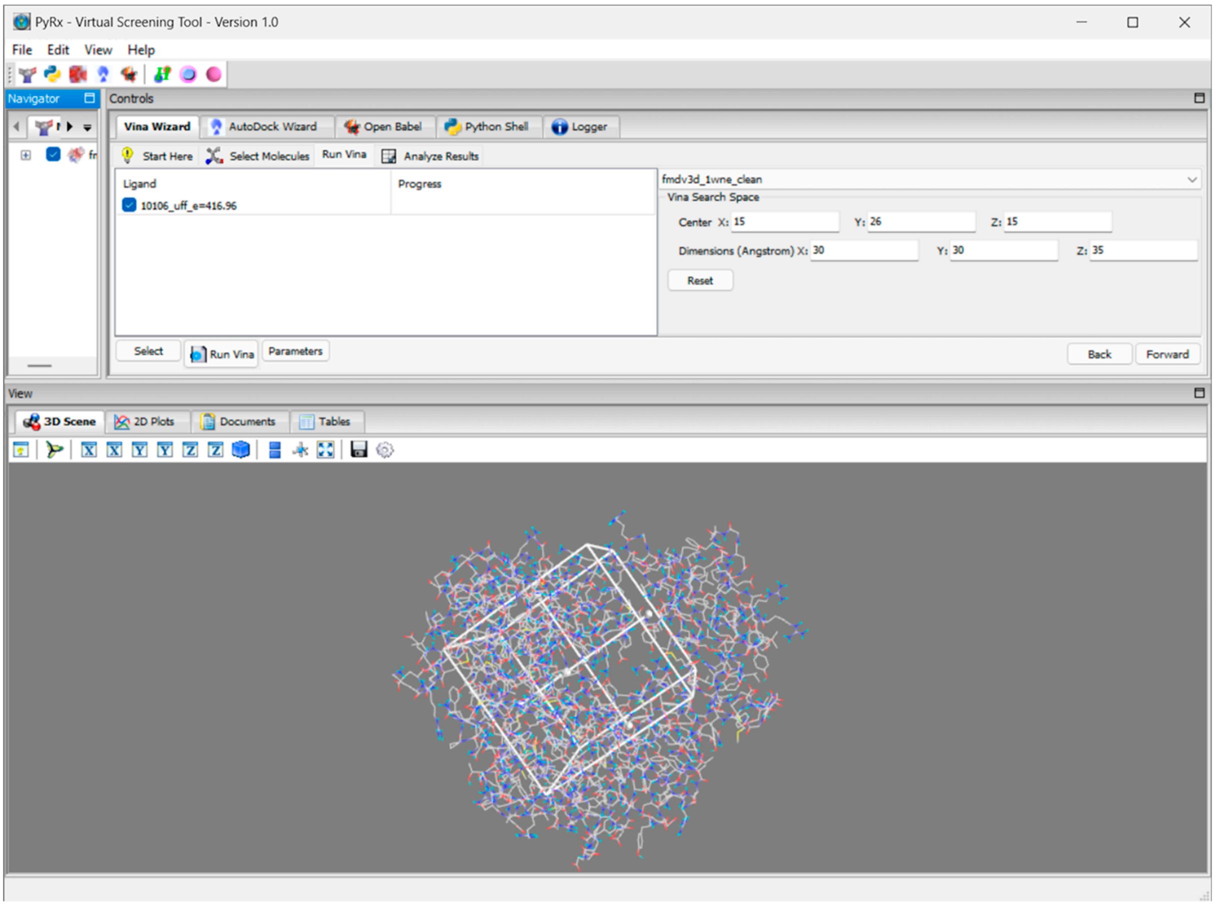

Figure S1. Parameters of the grid box and grid center used for virtual screening in PyRx software. The grid center was set at coordinates 15:26:15 (x:y:z) with a grid box size of 30 Å × 30 Å × 35 Å, which effectively covered the active site pocket and associated residues of the FMDV 3Dpol.

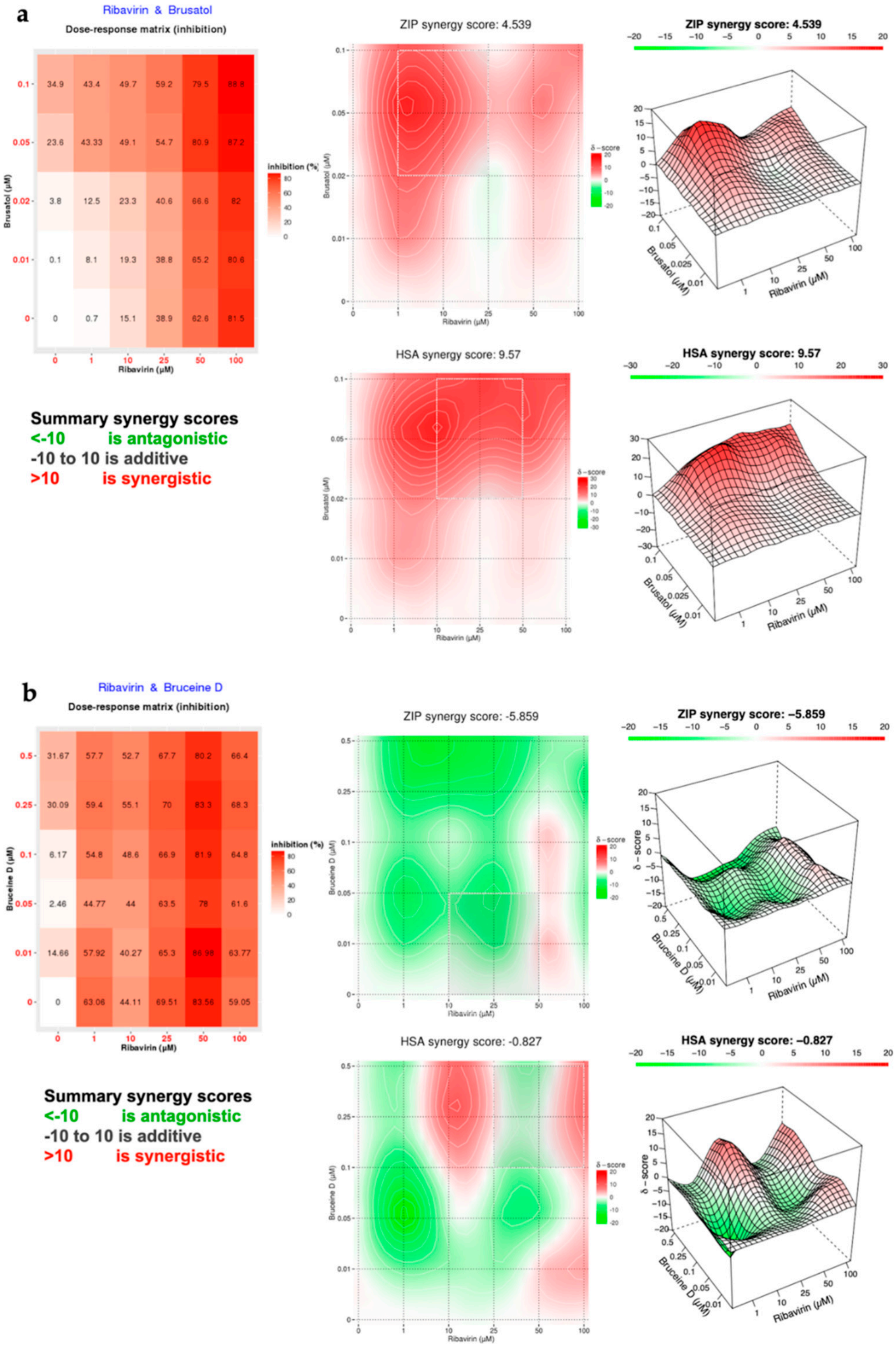

Figure S2. Interaction effects of the combination of either brusatol or bruceine D and the drug control, ribavirin, were analyzed using SynergyFinder version 3.0. The interaction scores for each method are depicted for (a) brusatol with ribavirin and (b) bruceine D with ribavirin. Summary synergy scores are interpreted as follows: <−10 is antagonistic, −10 to 10 is additive, and >10 is synergistic.

a

b

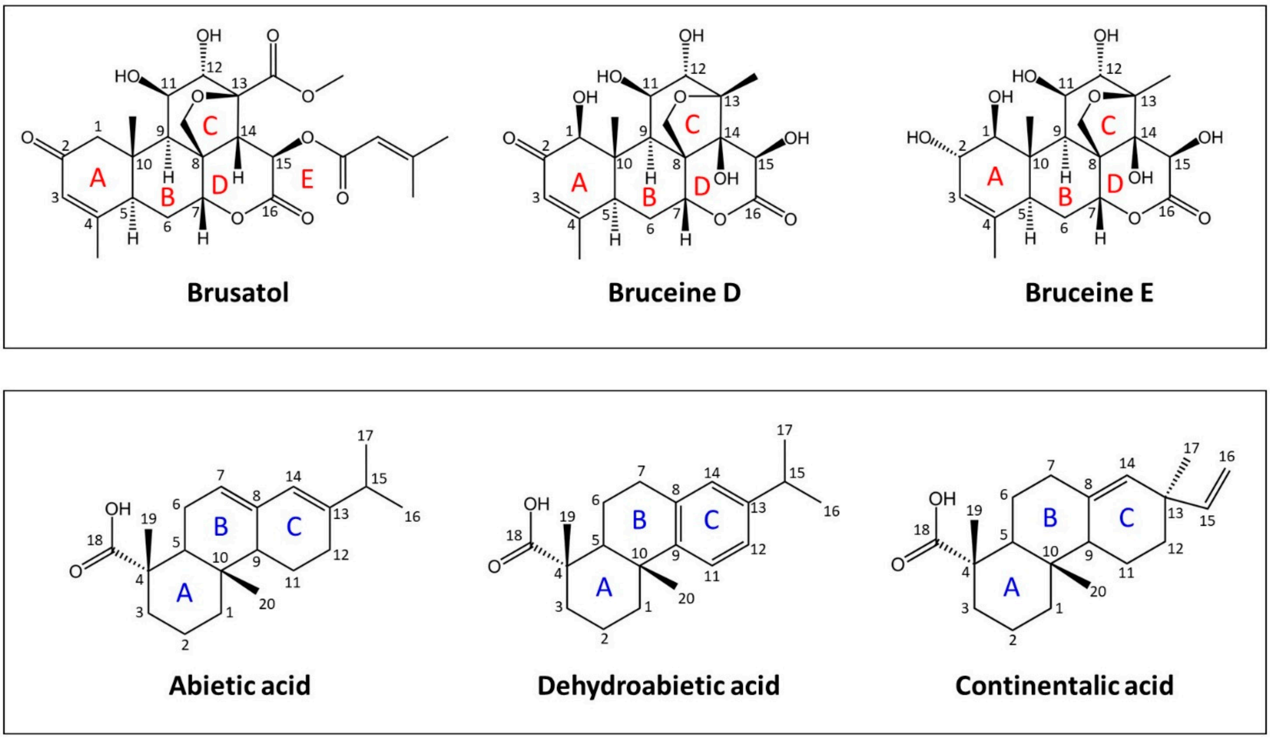

Figure S3. Chemical structures of (a) tetracyclic triterpenoids (brusatol, bruceine D, bruceine E) and (b) abietane diterpenoids (abietic acid, dehydroabietic acid, and continentalic acid).

Table S1. Structural-based virtual screening and physicochemical properties of top-ranked 32 terpenoids.

Rank	Name	Molecular formulas	Binding affinity (kcal/mol)	Mw (Dalton, Da)	cLogP	H-acceptors	H-donors	Total surface area (Angstrom, Å)	drug likeness
1	Saikosaponin B2	$C_{42}H_{68}O_{13}$	-10.5	780.98	1.78	13	9	540.21	-10.97
2	Limonin	$C_{26}H_{30}O_{8}$	-9.9	470.50	1.03	8	0	315.24	-3.00
3	Rosamultin	$C_{36}H_{58}O_{10}$	-9.4	650.85	2.49	10	7	449.03	-2.09
4	Tenuifolin	$C_{36}H_{56}O_{12}$	-9.2	680.37	1.17	12	8	463.63	-8.47
5	Ziyuglycoside II	$C_{35}H_{56}O_{8}$	-9.2	604.80	3.94	8	5	425.09	-4.06
6	Rutaevin	$C_{26}H_{30}O_{9}$	-9.1	486.51	0.18	9	1	320.33	-2.16
7	Cucurbitacin IIb	$C_{30}H_{48}O_{7}$	-8.4	520.70	2.93	7	5	369.56	-0.24
8	Ganoderic acid A	$C_{30}H_{44}O_{7}$	-8.3	516.70	3.69	7	3	371.25	-4.08
9	Ginkgolide A	$C_{20}H_{24}O_{9}$	-8.2	408.40	-1.07	9	2	242.06	-1.78
10	Brusatol	$C_{26}H_{32}O_{11}$	-8.2	520.53	-0.05	11	3	344.99	-6.55
11	Ginsenoside Rg3	$C_{42}H_{72}O_{13}$	-8.1	785.02	2.97	13	9	555.85	-6.26
12	Triptolide	$C_{20}H_{24}O_{6}$	-8.1	360.40	-0.11	6	1	237.43	0.62

13	Bruceine D	$C_{20}H_{26}O_9$	-8.1	410.40	-2.12	9	5	251.40	-3.06
14	Bruceine E	$C_{20}H_{28}O_9$	-7.9	412.43	-2.27	9	6	252.50	-1.89
15	Ginsenoside Rh1	$C_{36}H_{62}O_9$	-7.8	638.88	3.95	9	7	459.13	-4.65
16	Alisol F	$C_{30}H_{48}O_5$	-7.7	488.70	4.26	5	3	349.67	-2.23
17	Euphorbia factor L2	$C_{38}H_{42}O_9$	-7.5	642.70	6.74	9	0	478.13	-2.90
18	Forskolin	$C_{22}H_{34}O_7$	-7.3	410.50	1.32	7	3	288.98	-4.68
19	Jolkinolide B	$C_{20}H_{26}O_4$	-7.2	330.42	2.26	4	0	227.95	-3.29
20	Euphorbiasteroid	$C_{32}H_{40}O_8$	-7.2	552.66	4.46	8	0	411.21	-3.63
21	Triptophenolide	$C_{20}H_{24}O_3$	-7.0	312.40	3.69	3	1	230.77	-0.18
22	Oridonin	$C_{20}H_{28}O_6$	-6.9	364.43	0.59	6	4	231.86	-5.16
23	Ingenol	$C_{20}H_{28}O_5$	-6.9	348.44	0.91	5	4	234.86	-0.50
24	Dehydroabietic acid	$C_{20}H_{28}O_2$	-6.8	300.40	4.44	2	1	234.02	-3.70
25	Pseudolaric acid B	$C_{23}H_{28}O_8$	-6.8	432.46	2.14	8	1	317.84	-2.03
26	Lathyrol	$C_{20}H_{30}O_4$	-6.6	334.45	2.93	4	3	243.40	-3.81
27	10-Deacetylbaccatin III	$C_{29}H_{36}O_{10}$	-6.6	544.60	0.96	10	4	370.05	1.46
28	Kaurenoic acid	$C_{20}H_{30}O_2$	-6.5	302.46	4.12	2	1	223.44	-6.16

29	Rupestonic acid	$C_{15}H_{20}O_3$	-6.5	248.32	2.75	3	1	191.08	-4.35
30	Abietic acid	$C_{20}H_{30}O_2$	-6.4	302.45	4.30	2	1	236.44	-5.11
31	Simvastatin	$C_{25}H_{38}O_5$	-6.2	418.56	4.46	5	1	327.83	0.67
32	Continentalic acid	$C_{20}H_{30}O_2$	-6.0	302.50	4.33	2	1	235.68	-6.63

Table S2. Cytotoxicity level of terpenoids on BHK-21 cells using crystal violet staining.

Toxicity level	Compound names	Cytotoxic concentration; CC_{50} (μM)
**Non-toxic** (Cell viability 75 – 100%) 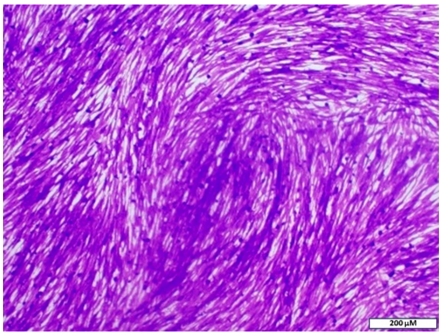	**Diterpenoids**	
	10-Deacetylbaccatin III	>500
	Continentalic acid	88.71 ± 1.82
	Euphorbiasteroid	>500
	Lathyrol	314.60 ± 2.50
	Rupestonic acid	72.29 ± 1.86
	Triptolide	122.40 ± 2.09
	**Triterpenoids**	
	Cucurbitacin IIb	240.50 ± 2.38
	Ganoderic acid A	>500
	Ginsenoside Rh1	173.60 ± 2.24
	Ginsenoside Rg3	269.90 ± 2.43
	Limonin	116.30 ± 2.07
	Rosamultin	333.20 ± 2.52
	Rutaevin	472 ± 2.67
	Saikosaponin B2	>500

Toxicity level	Compound names	Cytotoxic concentration; CC_{50} (μM)
**Mild** (Cell viability 50 – 75%) 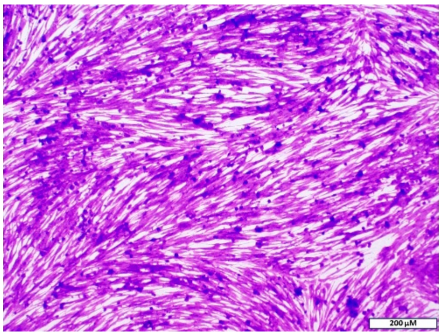	**Diterpenoids**	
	Abietic acid	99.25 ± 2.00
	Dehydroabietic acid	94.76 ± 1.98
	Euphorbia factor L2	114 ± 2.06
	Forskolin	75.94 ± 1.88
	Ginkgolide A	314.20 ± 2.50
	Ingenol	417.10 ± 2.62
	Jolkinolide B	46.51 ± 1.67
	Kaurenoic acid	266.10 ± 2.43
	Oridonin	46.75 ± 1.67
	Simvastatin	294.40 ± 2.47
	Triptophenolide	117.10 ± 2.07
	**Triterpenoids**	
	Alisol F	85.15 ± 1.93
	Bruceine E	49.65 ± 1.70
	Tenuifolin	54.94 ± 1.74
	Ziyuglycoside II	57.72 ± 1.76

Toxicity level	Compound names	Cytotoxic concentration; CC_{50} (μM)
**Moderate** (Cell viability 25 - 50%) 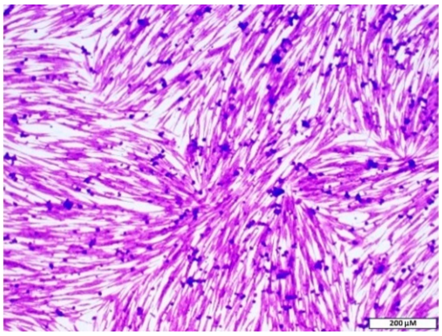	**Diterpenoids**	
	Pseudolaric acid	25.31 ± 1.40
	**Triterpenoids**	
	Bruceine D	4.51 ± 0.68
	Brusatol	18.54 ± 1.27
**DMSO** 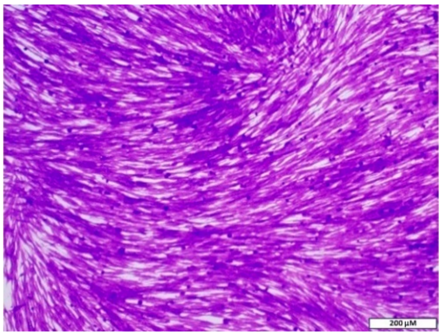	**Cell control** 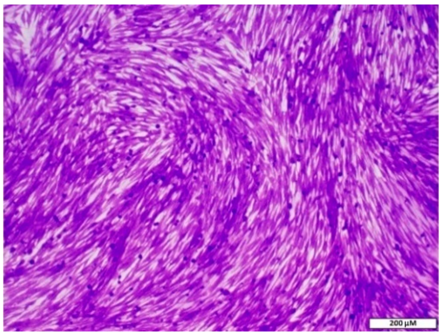	
